# Personal Profiles, Family Environment, Patterns of Smartphone Use, Nomophobia, and Smartphone Addiction across Low, Average, and High Perceived Academic Performance Levels among High School Students in the Philippines

**DOI:** 10.3390/ijerph18105219

**Published:** 2021-05-14

**Authors:** Danilo B. Buctot, Nami Kim, Sun-Hee Kim

**Affiliations:** Department of Addiction Science, Sahmyook University Graduate School, Seoul 01795, Korea; danny_bee2k3@yahoo.com (D.B.B.); sunnyisaac@naver.com (S.-H.K.)

**Keywords:** family environment, patterns of smartphone use, nomophobia, smartphone addiction, Filipino high school students, perceived academic performance

## Abstract

(1) Background: Problematic smartphone use in adolescents has become a major concern among parents and educators. This study aimed to determine the factors associated with, and the predictors of, low, average, and high perceived academic performance (PAP). (2) Methods: Descriptive and comparative analyses were employed in this cross-sectional study among 3374 Filipino high school students through an online Google forms survey. (3) Results: We found that age, grade level, father’s education, time spent daily on weekends, frequency of use on weekdays, purpose of use, nomophobia (NMP), and smartphone addiction (SA) were significantly associated with low PAP, while frequency of use on weekends and type of internet access had a significant association with high PAP. Gender was a significant predictor of low, average, and high PAP. Father’s education and SA were also significant predictors for both low and average PAP. (4) Conclusions: This study shows the significant association between personal profiles, family environment, patterns of smartphone use, NMP, and SA contributing to a significant impact on Filipino high school students’ PAP. This suggests that proper guidelines for smartphone use should be provided at home and in school settings to raise awareness of the adverse effects of SA on students’ academic performance.

## 1. Introduction

### 1.1. Smartphone Use, Nomophobia, Smartphone Addiction, and Academic Performance

Although smartphones are now considered a necessity for everyone, their improper use is becoming problematic and a major concern among adolescents’ parents and educators. Over the past decade, many studies have discussed the negative effects of smartphone addiction (SA) on individuals’ mental health and well-being [1] as well as on adolescents’ psychological well-being [2,3,4,5]. More recent studies have also pointed out the negative effects of smartphone use on students’ academic performance [1,6,7,8,9,10,11,12,13,14,15,16,17].

Studies that deal with the effects of smartphone use on academic performance have been conducted from various perspectives. Some have examined learning activities through smartphone use and found a lower grade point average (GPA) and cumulative GPA (CGPA) among students who often used their smartphones’ for learning [18,19], whereas others have examined different smartphone functions’ effect on academic performance [6]. Additionally, other works have determined the associations of time spent on smartphones [14], task-technology fit (TTF) [20], students’ self-control [21], behavioral intention in using smartphones [22], personal traits and mobile activities [23], fear of missing out (FOMO) [16], social media use [24], nomophobia (NMP), and SA [25] with academic performance.

As such, it seems that smartphone use can be a threat to students’ scholastic performance, particularly when the use of it becomes excessive [11], as this can lead to NMP and SA, which have impulsive, uncontrollable desires to use a smartphone as their common attribute. If someone becomes anxious when he/she is away from his/her smartphone, or if they fear losing it or not being able to access it, they are likely to be experiencing NMP [26,27,28,29]. On the other hand, when an individual manifest a strong urge to use a smartphone despite its negative effect (i.e., negligence of other aspects in life due to constant cravings and excessive use) [30,31,32,33], he or she is likely to be suffering from SA. NMP and SA are two modern behaviors that are highly prevalent in high school students [34], and this addictive tendency often manifests [35] as problematic use of smartphones [36].

### 1.2. Nomophobia and Smartphone Addiction in the Philippines

Smartphone use has become a trend among Filipino adolescents over the past few years. In fact, adolescents and young adults (16–24 years old) have the largest smartphone ownership percentage in the country [37]. According to Statista (The Statistics Portal), in the second and third quarter of 2018, Filipinos spent an average of 10.3 h a day online on their smartphones [38]. In 2020, a study reported an SA prevalence rate of 62.6% among Filipino adolescents [39]. Similarly, NMP among Filipino adolescents is also evident, as a local newspaper (Philippine Daily Inquirer) recently stated that 33.3% (i.e., one out of three) of Filipinos reported not being able to survive without their smartphones [40].

By 2003, cellphone use was already common among Filipino children and adolescents. The Department of Education Culture and Sports (DECS), which is currently referred to as the Department of Education (DepEd), was so concerned about students’ misguided and problematic activities that it issued orders (DECS orders Nos. 26 s. 2000 and 70 s. 1999–30 November 2003) prohibiting students of elementary and secondary schools from using cellphones during class. When smartphone use continued to dominate over the next few years, the DepEd reiterated the same orders (DepEd order No. 83 s. 2003) to remind school educators about the policy [41].

### 1.3. Personal Profiles, Family Environment, Patterns of Smartphone Use, Nomophobia, Smartphone Addiction, and Academic Performance

Patterns of smartphone use were found to be associated with NMP and SA, and these associations differed depending on demographics such as age, gender, and family environment. For instance, a previous study found age to be negatively associated with addictive behavior in smartphone use [42]. Furthermore, females manifested a greater likelihood of spending more time on their phones than males [43]. Additionally, people with lower education levels [3] or in lower age brackets [44] were more likely to manifest symptoms of SA.

Previous studies also claimed that NMP and SA are closely associated with each other [25,45,46], signifying that factors associated with NMP can also be factors of SA. For instance, [25] observed that adolescents with SA also showed nomophobic behaviors, and later found a positive correlation between nomophobic behaviors and social media addiction. Adolescents’ family environments also play a very important role in shaping their behaviors towards smartphone use [47]. A study among Korean adolescents found that SA is significantly associated with two-parent and double-income households as well as dysfunctional families exposed to domestic violence and parental addiction [47]. Additionally, family environments where adults frequently use their mobile devices could also lead to increased smartphone use among youngsters [48].

With regards to academic performance, it was found that having a strong family background and good education facilities could help enhance students’ performance [49]. Furthermore, parents’ educational background was also found to be associated with students’ academic performance [50], and broken families were significantly associated with lower academic achievement; these findings suggest that family structure is a significant factor in adolescents’ perceived academic performance (PAP) [51]. In addition, family size was found to be associated with low academic performance among students and with parents from low income households who struggle to pay school fees [49].

In a study on high school students, [52] determined the duration of smartphone ownership to be one of the influencing factors for NMP. In addition, [53] found a significant association of NMP with age, gender, duration, and frequency of smartphone use, social network sites (SNS) use, checking smartphones for no reason, and checking smartphones directly after waking up in the morning. Similarly, [25] identified gender, parents’ education levels, information and communication technology (ICT) use levels, duration and frequency of smartphone use, purpose, smartphone experience, and academic achievement as being significant predictors of both NMP and SA. Additionally, previous studies found high frequency and duration of smartphone use akin to SA severity [54,55], and the duration of SNS use and frequency of phone calls and text messages were found to be predictors of mobile phone addiction [43]. Other studies pointed out mental factors such as self-esteem, extraversion, conscientiousness, and emotional stability as significant predictors of NMP [56].

Thus, most previous studies agree with the notion that demographics (i.e., personal profiles and family environment) and patterns of smartphone use are significantly associated with NMP and SA and can negatively affect students’ academic performance. However, most previous studies have examined the adverse effects of the above-mentioned variables on academic performance based on students’ GPAs, while studies determining students’ self-PAP in relation to these factors remain scarce. To address these gaps, this study employed a sample of Filipino adolescents (i.e., junior and senior high school students) to investigate the association and the predictive capacity of personal profiles, family environment, patterns of smartphone use, NMP, and SA on students’ PAP.

### 1.4. Academic Performance and Perceived Academic Performance

Academic performance is measured by evaluating how much has been achieved over a certain period of time [57]. The most common and easy way to evaluate a student’s academic performance is by simply determining their GPA, whereby a higher GPA score indicates higher academic performance [58]. Previous studies note that GPA is a significant predictor of academic achievement [59,60]. However, GPA is a representation of academic achievement on a single unidimensional scale and is constructed entirely from course grade information; therefore, it is non-inclusive [61]. In contrast, PAP is a self-evaluation of academic performance that helps us understand how students view their academic achievement (i.e., high, average, low) and how they perceive themselves (i.e., positively or negatively), which relates to their self-esteem [62]. In this study, we asked participants to evaluate their academic performance based on how they perceive the effect of smartphone use on their academic grades.

### 1.5. Conceptual Framework

**Figure 1:**
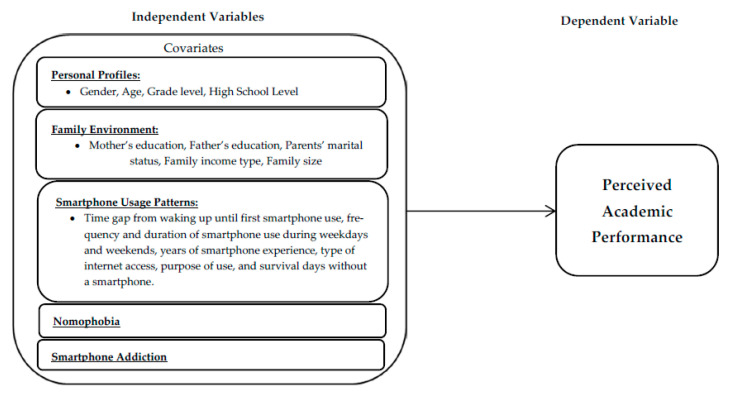
Research model of the study.

The concept of social cognitive theory is the basis of this study’s framework. Social cognitive theory points out that the mutual interaction of one’s personal factors, behavior, and environment influences future behavioral performance [63]. This, in turn, relates to behaviorism and the psychodynamic theory [25], in which people with smartphone use disorder believe they can escape negative emotions [25] such as loneliness and shyness [26] through smartphone use. Thus, according to [64], problematic smartphone use combines personal, cultural, environmental, and emotional factors. As the purpose of this study is to examine whether personal profiles (i.e., demographics), family environment (environmental), patterns of smartphone use, NMP, and SA (behavioral) are associated with PAP, we hypothesize that gender, age, grade level, high school level, parents’ education and marital status, family income, family size, duration of time from waking up until first smartphone use, duration and frequency of smartphone use on weekdays and weekends, years of smartphone experience, type of internet access, purpose of use, survival days without a smartphone, NMP, and SA significantly predict low, average, and high PAP. Figure 1 shows the research model of this study including the variables details.

## 2. Materials and Methods

### 2.1. Participants and Design

This cross-sectional method used convenience sampling to gather the data, which were collected from 11 schools in the Philippines. Participants were limited to Filipino high school students aged between 13 and 18 years old (i.e., from grades 7 to 12) who attended school at the time the study was conducted (during the 2019–2020 academic year, which started in July 2019 and ended in February 2020). A total of 3374 junior and senior high school students voluntarily participated in this study through an online Google Forms survey.

### 2.2. Procedure

Participants were provided with an informed consent form for them to sign after reading and understanding the purpose of the study, in which they were also assured that their responses would be confidential. After obtaining permission from the school administrators to use the school facility (i.e., computers in the computer rooms), the online survey was immediately launched, with simple tokens (i.e., chocolates, snacks, or candy) given immediately after the survey.

### 2.3. Materials

#### 2.3.1. Demographics (Personal Profiles and Family Environment), Smartphone Usage Questionnaire, and Perceived Academic Performance

This questionnaire includes demographic information (i.e., personal profiles) such as gender, age, grade level, high school level, parents’ education, family size, family income, and patterns of smartphone use (including the time gap from waking up until first smartphone use, frequency and duration of smartphone use during weekdays and weekends, years of smartphone experience, type of internet access, purpose of use, and survival days without a smartphone). Moreover, the participants were evaluated on whether they believed their smartphone use affected their academic performance by being asked whether their grades were “low,” “average” or “high” because of smartphone use. Thus, the students’ self-PAP was taken as the dependent variable.

#### 2.3.2. Nomophobia (NMP-Q Scale)

Developed in 2015, the NMP-Q scale consists of 20 questions pertaining to the following four dimensions: not being able to communicate (6 items), not being able to access information (4 items), losing connectedness (5 items), and giving up convenience (5 items). Responses to each item were recorded on a 7-point Likert scale that ranged from 1 (strongly disagree) to 7 (strongly agree) [29]. Total scores were computed and classified as follows: absence of NMP (20), mild level of NMP (21–59), moderate level of NMP (60–99), and severe level of NMP (100–140). In this study, Cronbach’s alpha was 0.916, and the following coefficients emerged for its subscales: not being able to communicate = 0.857, losing connectedness = 0.838, not being able to access information = 0.697, and giving up convenience = 0.743.

#### 2.3.3. Smartphone Addiction Scale-Short Version

The short version of the SA scale (SAS-SV) [3,65] was used to measure participants’ SA. Measured on a 6-point Likert scale, the answers of this 10-item questionnaire ranged from 1 (strongly disagree) to 6 (strongly agree) [3,65]; the cut-off scores were 31 and 33 for boys and girls, respectively [65]. The reliability score for this scale was a Cronbach’s alpha of 0.831.

### 2.4. Statistical Analyses

All analyses in this study were conducted using SPSS version 23 (SPSS Inc., Chicago, IL, USA) for Windows [66]. The main statistical method used in this study was a multiple linear regression analysis in which the PAP groups (i.e., low, average, and high) were the dependent variables, and the variables for personal profiles (i.e., age, gender, grade level, and high school level), family environment (i.e., parents’ education and marital status, family income type, and family size), and patterns of smartphone use (i.e., the time gap from waking up until first smartphone use, frequency and duration of smartphone use on weekdays and weekends, years of smartphone experience, type of internet access, purpose of use, and survival days without a smartphone) were the covariates. Additionally, a Pearson correlation analysis was used to explore the relationships between the variables, and a chi-square and one-way ANOVA were used to explore the differences between students across low, average, and high PAP groups.

Preliminary tests were conducted to ascertain that a multiple linear regression analysis was a good fit for this study and to ensure the validity of the data. The minimum number of cases per independent variable (20 cases) [67] was satisfied. Tests for normality (i.e., kurtosis and skewness) were not applied, as this study consisted of a very large sample [68]. With regards to multicollinearity issues, no scores close to zero and 10 were manifested for tolerance or variance inflation factor (VIF), which implied that there were no multicollinearity issues [69] in this study.

## 3. Results

### 3.1. General Characteristics of Variables

Table 1 shows the frequency, percentage, mean, and standard deviation of all the variables included in this study. The majority of participants were female (*n* = 1967, 58.3%) and around 60% (*n* = 2036, 60.3%) were junior high school students. The mean age of participants was 14 (M = 14.76, SD = 1.60) years old and the mean grade level was ninth grade (M = 9.16, SD = 1.50). Less than 1% (*n* = 17, 0.5%) did not have NMP. However, 15.9% (*n* = 537), 60.9% (*n* = 2055), and 22.7% (*n* = 765) had mild, moderate, and severe NMP, respectively. Thus, 95.5% (*n* = 3357) of the participants were classified as having NMP to some degree [(41.6% (*n* = 1395) males; 58.4% (*n* = 1962) females; (60.4% (*n* = 2026) juniors; (39.6% (*n* = 1331) seniors)], and 0.5% (*n* = 17) were classified as not having NMP. Moreover, 62.4% (*n* = 2105) of participants had SA ((42.3% (*n* = 890) males; 57.7% (*n* = 1215) females; (60.8% (*n* =1279) juniors; (39.2% (*n* =826) seniors)), and 37.6% (*n* = 1269) were normal smartphone users who did not have SA.

The majority of participants’ parents had received up to a “high school” education (Mother (*n* = 1331, 39.4%); Father (*n* = 1275, 37.8%)), followed by “Bachelor’s degree” (Mother (*n* = 868, 25.7%); Father (*n* = 837, 24.8%)), and “Associate degree” (Mother (*n* = 534, 15.8) %); Father (*n* = 538, 15.9%)). Furthermore, the majority (71.6%) of participants’ parents were “legally married” with an average family size of 5.53 people, and over half (55.3%) reported coming from double-income households.

This study also reported that 42.1% (*n* = 1422) of participants had been using smartphones for the last 3 to 6 years, with an average of 4.90 years of smartphone experience. The largest portion of time gap from waking up until the first smartphone use was within 5 min, as reported by 40.5% of participants (*n* = 1367). Furthermore, participants spent an average of 8.87 and 9.91 h a day on their smartphones during the week and weekends, respectively. Concerning the frequency of use, participants used their smartphones 20 times or less at an average frequency of 8.98 and 10.24 times a day on weekdays and weekends, respectively. With regard to the type of internet access, 54.4% of participants reported having “Wi-Fi,” while 27.5% indicated using a “prepaid internet card.” The top three purposes of smartphone use were accessing social network sites (SNS) (*n* = 1700, 50.4%), online chatting (*n* = 562, 16.7%), and playing games (*n* = 382, 11.3%). When asked how many days they could survive without a smartphone, 59.7% (*n* = 2105) of participants indicated “three days or less.” Lastly, when participants were asked how their smartphone use affected their academic grades, 60% (*n* = 2023) indicated that their grades were “average,” while 23% (*n* = 777) and 17% (*n* = 574) answered “high” and “low,” respectively.

### 3.2. Association between Personal Profiles, Patterns of Smartphone Use, Nomophobia, Smartphone Addiction, and Perceived Academic Performance

Table 2 indicates that low PAP was significantly correlated with gender (*r* = −0.039; *p* < 0.05), age (*r* = −0.064; *p* < 0.01), grade level (*r* = −0.087; *p* < 0.01), high school level (*r* = −0.088; *p* < 0.01), mother’s education (*r* = 0.056; *p* < 0.01), father’s education (*r* = 0.065; *p* < 0.01), time spent daily on weekends (*r* = −0.042; *p* < 0.05), frequency of smartphone use on weekdays (*r* = −0.039; *p* < 0.05), years of smartphone experience (*r* = −0.051; *p* < 0.01), purpose of use (*r* = 0.042; *p* < 0.05), NMP (*r* = 0.058; *p* < 0.01), and SA (*r* = 0.116; *p* < 0.01).

Furthermore, high PAP was significantly correlated with gender (*r* = −0.050; *p* < 0.01), high school level (*r* = −0.034; *p* < 0.05), mother’s education (*r* = −0.054; *p* < 0.01), frequency of use on weekends (*r* = 0.046; *p* < 0.01), years of smartphone experience (*r* = 0.055; *p* < 0.01), and type of internet access (*r* = −0.048; *p* < 0.01).

### 3.3. Differences in Personal Profiles, Family Environment, Patterns of Smartphone Usage, Nomophobia, and Smartphone Addiction across Different Academic Performance Groups

Table 3 reveals that there was a significant difference between low, average, and high PAP in terms of gender (*X*^2^ = 18.05, *p* = 0.000), age (*F* (5, 3368)= 3.46, *p* = 0.004), grade level *F* (5, 3368)= 4.70, *p* = 0.000), high school level (*X*^2^ = 26.74, *p* = 0.000), mother’s education (*X*^2^ = 26.34, *p* = 0.023), father’s education (*X*^2^ = 35.49, *p* = 0.001), daily time spent using smartphones on weekdays (*F* (49, 3324) = 1.55, *p* = 0.009), frequency of use of smartphones on weekdays (*F* (62, 3311) = 1.39, *p* = 0.024), years of smartphone experience (*F* (12, 3361) = 3.82, *p* = 0.000, type of internet access (*X*^2^ = 12.63, *p* = 0.049), survival days without a smartphone (*F* (73, 3300) = 1.44, *p* = 0.009), and SA (*F* (50, 3323) = 1.68, *p* = 0.002).

### 3.4. Predicton Factors of Low, Average, and High Perceived Academic Performance

Table 4 shows the results of the multiple linear regression analysis predicting low, average, and high PAP. Gender (*β* = −0.044; *p* < 0.05), age (*β* = 0.073; *p* < 0.05), grade level (*β* = −0.097; *p* < 0.05), father’s education (*β* = 0.044; *p* < 0.05), frequency of use on weekdays (*β* = −0.111; *p* < 0.05), purpose of use (*β* = 0.048; *p* < 0.01), survival days without a smartphone (*β* = 0.063; *p* < 0.01), and SA (*β* = 0.126; *p* < 0.001) were significant predictors of low PAP. On the other hand, high PAP was significantly predicted by gender (*β* = −0.048; *p* < 0.01), family size (*β* = 0.064; *p* < 0.05), type of internet access (*β* = −0.035; *p* < 0.05), and survival days without a smartphone (*β* = −0.055; *p* < 0.05), while gender (*β* = 0.075; *p* < 0.05), father’s education (*β* = −0.054; *p* < 0.05), family size (*β* = −0.054; *p* < 0.05), type of internet access (*β* = 0.054; *p* < 0.01), and SA (*β* = −0.101; *p* < 0.001) were also significant predictors of average PAP.

## 4. Discussion

The purpose of this study was three-fold. First, it aimed to determine the factors that are associated with PAP. Second, it explored the differences between students across low, average, and high PAPs with regard to their personal profiles, family environment, patterns of smartphone use, NMP, and SA. Finally, this study examined the predictive factors of low, average, and high PAP.

We found that low and high PAP scores were significantly associated with gender, high school level, mother’s education, and years of smartphone experience. Age, grade level, father’s education, daily time spent using smartphones on weekends, frequency of use of smartphones on weekdays, purpose of use of smartphones, NMP, and SA were significantly associated with low PAP, whereas frequency of use on weekends and type of internet access were revealed to have a significant association with high PAP. The results suggest that PAP scores are influenced by these variables. A previous study found that more mature university students (i.e., those aged 23 and over) scored higher in Revised Approaches to Studying Inventory (RASI) orientation than those in lower age groups [70], thereby suggesting an increase in academic performance with an increase in age. Our finding of a significant association between age, grade level, and low PAP coincides with other papers’ findings that high school students’ age has a significant effect on their academic performance [71]. Moreover, our study supports previous findings that parents’ educational backgrounds are correlated with students’ academic performance [50], which can be positively influenced by both family income (i.e., father’s income) and parents’ education levels [49].

It is interesting to note that this study found that there was a significant negative association between daily time spent on smartphones on weekends (i.e., 10 h on average), the frequency of use on weekdays (i.e., 20 times or less each day), and low PAP. Moreover, high PAP was significantly and positively associated with frequency of use on weekends (i.e., 20 times or less per day). These results indicate that the likelihood of having low PAP decreases as the duration of smartphone use on weekends and frequency of smartphone use on weekdays and weekends increase, which also signify that participants with high duration and frequency of smartphone use and longer experience with smartphone use are more likely to have better perceptions about their academic achievement; this is inconsistent with findings from a recent study on Chinese adolescents’ poor academic performance due to prolonged smartphone use [72].

It seems that for Filipino high school students, smartphone use gives them the confidence to perform well in their studies. For example, perhaps they used smartphone apps for learning, which could effectively enhance productivity and academic performance [73], as well as increase the likelihood of positively perceiving their academic improvement [22]. Applying the same logic, Singaporean university students who used smartphones for learning purposes reported having higher GPA scores [74]. In this study, accessing SNS or social media was the most common purpose of smartphone use. According to studies on SNSs [75,76], Twitter gave students more freedom to ask questions and have discussions that are helpful for enhancing students’ engagement and academic achievement [75]. Similarly, Facebook use helped promote co-curricular activities, which can lead to academic success and boost individual well-being [76].

Our findings also suggested that having more years of smartphone experience (i.e., 5 years on average) was positively associated with high PAP. This indicates the possibility of students with more years of smartphone experience or ownership having more positive perceptions about their academic performance. To the best of our knowledge, no study has examined this relationship so far. However, a study among Turkish high school students found a positive correlation between duration of smartphone ownership and FOMO [77]. As mentioned earlier, students these days use smartphones for academic learning purpose [73], thus the longer they own a smartphone, the more academic learning enhancement they benefit from; this, in turn, goes some way to explaining why they feel anxious when smartphones are inaccessible. Furthermore, we found that the purpose of use (i.e., SNS) was associated with low PAP. This suggests that the use of SNS as the purpose of smartphone use is associated with students’ poor perceptions of academic performance. This is in line with previous findings stating that the addictive use of social media leads to low self-esteem or negative self-evaluation [78]. Additionally, studies in the past noted that SNS use distracts students’ cognition and affects academic performance [79,80].

Furthermore, NMP and SA were found to be positively associated with low PAP, thereby suggesting that students perceive their academic performance to be poorer as their levels of NMP and SA increase. These findings are consistent with the results of a systematic review about the adverse effects of NMP on academic performance [81] and a study among nomophobic university students that demonstrated weak academic performance [82]; similarly, a study among Turkish undergraduate students and other selected university students found that there was a negative relationship between academic performance and SA [1,16], as well as between students’ in-class smartphone use and academic grades [83]. As for adolescents and children, a study of screen-based activities (i.e., social media use) also found it to be negatively associated with academic performance [84].

We also found a significant difference between low, average, and high PAP in terms of gender, age, grade level, high school level, mother’s education, father’s education, time spent daily using smartphones on weekdays, frequency of use of smartphones on weekdays, years of smartphone experience, type of internet access, survival days without a smartphone, and SA. Surprisingly, participants in the high PAP group spent a significantly longer time using their smartphones during weekdays, and had higher frequency of smartphone use on weekdays, and longer years of smartphone use experience than those in the average and low PAP groups. Again, these findings indicate a better PAP when high school students consistently use smartphones for learning purposes, as discussed earlier [22,72,73,74,75,76]. In addition, participants in the low PAP group had significantly higher levels of smartphone addiction, which again confirms the positive association between SA and low PAP [1,16,83].

Participants in the high PAP group reported significantly lower survival days without a smartphone than those in low and average groups. The majority (60%) of the participants in this study indicated being able to survive for up to 3 days without their smartphones. This supports a recent survey in the Philippines, which reported that one out of three Filipinos could not survive without a smartphone [40]; Similar results were also found in the Australian context [85], which indicated that not being able to use a smartphone may lead to NMP [26,27,28,29] and FOMO [86].

Furthermore, we found that gender was a significant predictor of low, average, and high PAP, which is in line with previous findings that suggested that gender is an important factor in academic grades (whereby women are more motivated in terms of academic achievements than men) [87]. The education level of fathers and SA were also significant predictors for both low and average PAP. These are consistent with previous studies that found a significant relationship between parental educational levels and academic performance of students [88], as well as the possibility of fathers’ academic efficacy enhancing academic performance, especially for girls [89]. Other studies have also concluded that SA is a predictive factor for academic performance [16], and there is a significant association between problematic smartphone use and lower GPAs or worse academic performances [12].

In addition, family size and type of internet access were significant predictors of average and high PAP levels. This indicates that family size (i.e., average size of 5.53) and the way students access the internet influence their PAP. A study among Nigerian students found a significant relationship between family size and academic performance [88]. A study conducted among postsecondary level students also found that those who have access to websites at school (regardless of the type of access) and made use of the tools for e-learning performed better in their examinations [90]. Furthermore, age, grade, frequency of smartphone use on weekdays, and purpose of use were significant predictors of low PAP. In other words, high school students’ (who were at an average age and grade level of 14.76 and 9.16, respectively) perceptions of academic performance are significantly impacted when they use their smartphones at least 20 times a day on weekdays and primarily for SNS.

Another study was conducted examining the effect of TTF of smartphones on PAP and smartphone use among Korean college students [20], and it was found that the TTF of smartphones directly influenced the impact of students’ PAP and indirectly influenced their attitude toward smartphone use [20]. To the best of our knowledge, no studies have examined the impact of frequency and purpose of smartphone use on students’ PAP. Nonetheless, in a study among Turkish adolescents, school achievement was found to be a significant factor for problematic smartphone use or SA [25], suggesting that smartphone use impacts students’ academic performance. This confirms previous findings that there are significant associations between smartphone use and students’ exam results [9], frequency of smartphone use and academic success [7], and higher smartphone use and poor academic performance [14].

The purpose of smartphone use (i.e., SNS) significantly predicted low PAP in the sense that they were positively associated with each other, which is again consistent with previous findings that there was a negative association between social media use and academic performance [24,84]. Moreover, in a study among undergraduate students in Singapore, mobile phone activity (i.e., improper use of smartphones) was found to be a critical predictive factor that mediated the relationship between smartphone dependency and GPA scores [23]. However, [23] pointed out that the effect of social media use on students’ GPAs was not as bad as the effect of playing video games. Thus, personal traits (i.e., self-control and self-efficacy) help students to effectively handle smartphone use in order to achieve better academic performance [21] and to enhance their positive perceptions of their own academic performance [22].

## 5. Conclusions

This study clearly revealed the significant association between personal profiles, family environment, patterns of smartphone use, NMP, and SA, and students’ low and high PAP. Our findings suggest that Filipino high school students with high smartphone use perceived their academic performance to be better. However, given that frequency of smartphone use on weekdays, purpose of use, and SA significantly predict low PAP, we conclude that problematic use of smartphones impacts how Filipino high school students perceived their academic performance.

### 5.1. Implications

This study found a high percentage of NMP and SA rates among participants, which indicate a high level of smartphone dependence. Despite the likelihood of high PAP when smartphone use increases, proper guidelines on smartphone use should be provided at home and in school to raise awareness of the adverse effects of SA on students’ academic performance.

### 5.2. Limitations and Future Directions

Despite its contributions, this study has some limitations: first, as this is a cross-sectional study, it is difficult to determine the cause and effect relationship. To address this issue, it is recommended that a longitudinal research design should be employed. Second, this study uses a non-probability convenience sampling method in which we cannot generalize the results to the entire population. To ensure that the sample is representative of the overall population, a probability sampling method should be used. Third, our participants consisted of high school students. Future research should include a comparative study between NMP, SA, and PAP among participants with different levels of education (i.e., elementary, undergraduate, and graduate school students). Furthermore, data on patterns of smartphone use, such as duration and frequency of smartphone use on weekdays and weekends, were collected through self-reports. This raises the issue of sincerity and accuracy of the reported hours and frequency. To avoid this in future studies, a smartphone app should be used to keep record of the actual daily duration and frequency of smartphone use in order to obtain accurate smartphone usage data. Finally, PAP focuses more on how the participants evaluate their scholastic performance, which also paves the way for examining their self-esteem, and thus results cannot be compared with previous studies which used GPA to evaluate participants’ academic performance. Thus, in future studies it is recommended to use both PAP and overall GPA to evaluate the impact of demographics, patterns of smartphone use, NMP, and SA on students’ academic performance.

## Figures and Tables

**Table 1:** Frequency, percentage, mean, and standard deviation among variables.

Variables	*n* (M)	% (SD)	Variables	*n* (M)	% (SD)
Personal Profiles:			Time spent daily (on weekends):	(9.91) ^a^	(5.82) ^a^
Gender:			Below 4 h	285	8.4
Male	1407	41.7	4 to 6 h	671	19.9
Female	1967	58.3	7 to 9 h	799	23.7
Age:	(14.76)	(1.60)	10 h or more	1619	48.0
13	973	28.8	Frequency of use (on weekdays):	(8.98) ^a^	(14.50) ^a^
14	742	22	20 times or less	3164	93.8
15	683	20.2	21 to 40 times	114	3.4
16	357	10.6	41 to 60 times	46	1.4
17	352	10.4	61 to 80 times	11	0.3
18	267	7.9	81 times or more	39	1.2
Grade Level:	(9.16)	(1.50)	Frequency of use of smartphones (on weekends):	(10.24) ^a^	(27.09) ^a^
Junior high school:	2036	60.3	20 times or less	3131	92.8
7	508	15.1	21 to 40 times	113	3.3
8	754	22.3	41 to 60 times	68	2.0
9	774	22.9	61 to 80 times	9	0.3
Senior high school:	1338	49.7	81 times or more	53	1.6
10	691	20.5	Years of Smartphone Experience:	(4.90) ^a^	(2.70) ^a^
11	332	9.8	Below 3 years	1113	33.0
12	315	9.3	3 to 6 years	1422	42.1
Family Environment:			7 to 10 years	719	21.3
Mother’s Education:			11 years and above	120	3.6
PhD	101	3.0	Type of Internet Access:		
MA	289	8.6	Wi-Fi	1834	54.4
Bachelor’s	868	25.7	Prepaid Internet card	929	27.5
Associate	534	15.8	Monthly subscription	137	4.1
High School	1331	39.4	Other	474	14.0
Middle School	65	1.9	Purpose of Smartphone Use:		
Elementary	178	5.3	SNS	1700	50.4
Never studied	8	0.2	Phone Calls	121	3.6
Father’s Education:			Play Games	382	11.3
PhD	99	2.9	SMS	57	1.7
MA	294	8.7	Chatting online	562	16.7
Bachelor’s	837	24.8	Checking emails	13	0.4
Associate	538	15.9	Watching videos/movies	165	4.9
High School	1275	37.8	Listening to music	166	4.9
Middle School	81	2.4	Reading the news	20	0.6
Elementary	229	6.8	Taking pictures	15	0.4
Never studied	21	0.6	Other	173	5.1
Parents’ Marital Status:			Survival days without a smartphone:		
Legally Married	2417	71.6	3 days or less	2015	59.7
Separated	302	9.0	4 to 7 days	819	24.3
Divorced	38	1.1	8 to 11 days	97	2.9
Annulled	36	1.1	12 to 15 days	85	2.5
Single Parent	308	9.1	16 to 20 days	25	0.7
Widowed	0	0	21 to 25 days	24	0.7
Other	273	8.1	26 to 30 days	94	2.8
Family Size:	(5.53) ^a^	(2.73) ^a^	31 days or more	215	6.4
3 people or less	373	11.1	Nomophobia Group:		
4 to 6 people	2223	65.9	Without Nomophobia	17	0.5
7 to 9 people	608	18.0	With Nomophobia	3357	99.5
10 people or more	170	5.0	Nomophobia Level:		
Family Income Type:			Absence of nomophobia	17	0.5
Single Income household	1507	44.7	Mild nomophobia	537	15.9
Double Income household	1867	55.3	Moderate nomophobia	2055	60.9
Patterns of Smartphone Use:			Severe nomophobia	765	22.7
Time gap from waking up until first smartphone use:			Smartphone Addiction Group:		
Within 5 min	1367	40.5	Without smartphone addiction	1269	37.6
Within 6–30 min	1248	37.0	With smartphone addiction	2105	62.4
Within 31–60 min	336	10.0	Perceived Academic Performance:		
More than 60 min	423	12.5	Low	574	17
Time spent daily(on weekdays):	(8.87) ^a^	(11.78) ^a^	Average	2023	60
Below 4 h	416	12.3	High	777	23
4 to 6 h	904	26.8			
7 to 9 h	872	25.8			
10 h and above	1182	35.0			

^a^ = taken from the actual figures, provided by the respondents.

**Table 2:** Summary of socio-demographic variables, nomophobia, smartphone addiction, and perceived academic performance (Pearson correlation analysis).

	Personal Profiles				Family Environment					Smartphone Usage Patterns									D	E	F	G	H
Variables	A1	A2	A3	A4	B1	B2	B3	B4	B5	C1	C2	C3	C4	C5	C6	C7	C8	C9					
A. Personal Profiles:																							
1. Gender	−																						
2. Age	0.029	−																					
3. GL	0.081 **	0.883 **	−																				
4. HSL	0.059 **	0.767 **	0.846 **	−																			
B. Family Environment:																							
1. ME	0.120 **	0.036 *	0.060 **	0.029	−																		
2. FE	0.116 **	0.052 **	0.082 **	0.046 **	0.613 **	−																	
3. PMS	−0.039 *	−0.043 *	−0.073 **	−0.039 *	0.083 **	0.090 **	−																
4. FIT	−0.077 **	−0.025	−0.051 **	−0.024	−0.177 **	−0.150 **	−0.070 **	−															
5. FS	0.015	0.025	0.014	0.004	0.109 **	0.0930 **	−0.004	−0.018	−														
C. Smartphone Usage Patterns:																							
1. TGWFSU	−0.061 **	−0.045 **	−0.046 **	−0.006	0.004	−0.012	0.036 *	−0.012	0.002	−													
2. TSDWd	0.088 **	0.119 **	0.126 **	0.141 **	−0.020	−0.029	0.009	0.008	0.011	0.089 **	−												
3. TSDWe	0.124 **	0.136 **	0.138 **	0.151 **	−0.079 **	−0.084 **	−0.037 *	0.025	0.001	0.078 **	0.631 **	−											
4. FUWd	0.045 **	0.081 **	0.078 **	0.084 **	−0.101 **	−0.114 **	−0.066 **	0.019	−0.025	0.000	0.179 **	0.208 **	−										
5. FUWe	0.049 **	0.064 **	0.068 **	0.640 **	−0.079 **	−0.080 **	−0.057 **	0.021	−0.016	−0.001	0.110 **	0.162 **	0.633 **	−									
6. SYE	0.044 *	0.185 **	0.190 **	0.191 **	−0.120 **	−0.112 **	−0.016	0.027	−0.027	−0.004	0.216 **	0.212 **	0.135 **	0.098 **	−								
7. IAT	0.018	0.060 **	0.075 **	0.054 **	0.194 **	0.178 **	0.041 *	−0.088 **	0.009	−0.024	−0.064 **	−0.086 **	−0.051 **	−0.048 **	−0.104 **	−							
8. PU	−0.081 **	−0.038 *	−0.048 **	−0.053 **	0.006	−0.017	0.033	0.034 *	0.009	0.008	−0.052 **	−0.075 **	−0.018	−0.037 *	−0.021	0.025	−						
9. SDWS	−0.103 **	−0.012	−0.031	−0.037 *	−0.114 **	−0.092 **	0.000	0.069 **	−0.013	0.016	−0.020	−0.011	0.064 **	0.038 *	0.029	−0.028	0.016	−					
D. NMP	0.155 **	0.018	0.039 *	0.045 **	0.077 **	0.061 **	−0.015	−0.038 *	0.011	0.040 *	0.194 **	0.182 **	0.100 **	0.073 **	0.065 **	0.005	−0.107 **	−0.114 **	−				
E. SA	0.097 **	−0.028	0.021	0.002	0.081 **	0.084 **	−0.003	−0.025	0.005	0.031	0.211 **	0.185 **	0.059 **	0.031	0.027	0.009	−0.106 **	−0.084 **	0.642 **	−			
F. Low PAP	−0.039 *	−0.064 **	−0.087 **	−0.088 **	0.056 **	0.065 **	0.014	−0.033	0.006	−0.011	0.002	−0.042 *	−0.039 *	−0.015	−0.051 **	0.014	0.042 *	0.021	0.058 **	0.116 **	−		
G. Average PAP	0.073 **	0.024	0.039 *	0.038 *	0.003	−0.028	0.007	0.001	0.001	−0.009	−0.015	0.014	−0.010	−0.015	−0.008	0.052 **	−0.0015	−0.005	−1.025	−0.078 **	−0.554 **	−	
H. High PAP	−0.050 **	0.030	0.032	0.034 *	−0.054 **	−0.026	−0.020	0.028	−0.007	0.021	0.016	0.021	0.025	0.046 **	0.055 **	−0.048 **	−0.020	−0.014	−0.022	−0.013	−0.248 **	−0.669 **	−

Note. GL = Grade Level; HSL = High School Level; ME = Mother’s Education; FE = Father’s Education; PMS = Parents’ Marital Status; FIT = Family Income Type; FS = Family Size; TGWFSU = Time Gap from Waking up until first smartphone use; TSDWd = Time Spent Daily on Weekdays; TSDWe = Time Spent Daily on Weekends; FUWd = Frequency of Use on Weekdays; FUWe = Frequency of Use on Weekends; SYE = Years of Smartphone Experience; PU = Purpose of Use; IAT = Type of Internet Access; SDWS = Survival Days Without a Smartphone; NMP = Nomopobia; SA = Smartphone Addition; PAP = Perceived Academic Performance; * *p* < 0.05, ** *p* < 0.01.

**Table 3:** Differences in personal profiles, family environment, smartphone usage pattern, smartphone addiction, and nomophobia across perceived academic performance groups.

Variables	Low PAP (*n* = 574)		Average PAP (*n* = 2023)		High PAP (*n* = 777)		F (*X*^2^)	*p*
	*n* (M)	% (SD)	*n* (M)	% (SD)	*n* (M)	% (SD)		
Personal Profiles:								
Gender:							(18.05)	0.000
Male	264	46	784	38.8	359	46.2		
Female	310	54	1239	61.2	418	53.8		
Age	(14.53)	(1.51)	(14.79)	(1.58)	(14.84)	(1.67)	3.46	0.004
Grade level	(8.87)	(1.42)	(9.21)	(1.49)	(9.25)	(1.56)	4.70	0.000
High school level:							(26.74)	0.000
Junior	401	69.9	1190	58.8	445	57.3		
Senior	173	30.1	833	41.2	332	42.7		
Family Environment:								
Mother’s education:							(26.34)	0.023
PhD	15	2.6	61	3.0	25	3.2		
MA	40	7.0	177	8.7	72	9.3		
Bachelor’s	125	21.8	519	25.7	224	28.8		
Associate	85	14.8	319	15.8	130	16.7		
High School	260	45.3	790	39.1	281	36.2		
Middle School	14	2.4	35	1.7	16	2.1		
Elementary	34	5.9	118	5.8	26	3.3		
Never studied	1	0.2	4	0.2	3	0.4		
Father’s education:							(35.49)	0.001
PhD	13	2.3	64	3.2	22	2.8		
MA	49	8.5	165	8.2	80	10.3		
Bachelor’s	120	20.9	518	25.6	199	25.6		
Associate	81	14.1	327	16.2	130	16.7		
High School	236	41.1	767	37.9	272	35.0		
Middle School	9	1.6	55	2.7	17	2.2		
Elementary	61	10.6	119	5.9	49	6.3		
Never studied	5	0.9	8	0.4	8	1.0		
Parents’ marital status:							(8.54)	0.576
Legally Married	403	70.2	1440	71.2	574	73.9		
Separated	52	9.1	188	9.3	62	8.0		
Divorced	7	1.2	18	.9	13	1.7		
Annulled	8	1.4	22	1.1	6	.8		
Single Parent	55	9.6	193	9.5	60	7.7		
Widowed	0	0	0	0	0	0		
Other	49	8.5	162	8.0	62	8.0		
Family income type:							(5.09)	0.078
Single income household	277	48.3	903	44.6	327	42.1		
Double income household	297	51.7	1120	55.4	450	57.9		
Family size:	(5.59) ^a^	(2.20) ^a^	(5.48) ^a^	(2.19) ^a^	(5.65) ^a^	(4.03) ^a^	(3.09)	0.798
3 people or less	62	10.8	229	11.3	82	10.6		
4 to 6 people	374	65.2	1329	65.7	520	66.9		
7 to 9 people	111	19.3	355	17.5	142	18.3		
10 people or more	27	4.7	110	5.4	33	4.2		
Patterns of Smartphone Use:								
Time gap from waking up until first smartphone use:							(7.95)	0.242
Within 5 min	251	43.7	810	40.0	306	39.4		
Within 6–30 min	197	34.3	768	38.0	283	36.4		
Within 31–60 min	47	8.2	206	10.2	83	10.7		
After more than 60 min	79	13.8	239	11.8	105	13.5		
Time spent per day (on weekdays)	(8.92)	(5.94)	(8.58)	(5.19)	(9.60)	(22.50)	1.55	0.009
Time spent per day (on weekends)	(9.70)	(7.08)	(9.94)	(5.63)	(9.99)	(5.26)	0.91	0.631
Frequency of use (on weekdays)	(7.74)	(12.00)	(8.88)	(13.15)	(10.17)	(18.83)	1.39	0.024
Frequency of use (on weekends)	(9.36)	(43.12)	(10.01)	(20.88)	(11.49)	(26.05)	1.17	0.174
Years of smartphone experience	(4.59)	(2.87)	(4.89)	(2.66)	(5.19)	(2.63)	3.82	0.000
Type of internet access:							(12.63)	0.049
Wi-Fi	325	56.6	1068	52.8	441	56.8		
Prepaid Internet card	147	25.6	558	27.6	224	28.8		
Monthly subscription	25	4.4	85	4.2	27	3.5		
Other	77	13.4	312	15.4	85	10.9		
Purpose of use:							(1.02)	0.426
SNS	250	43.6	1054	52.1	396	51.0		
Phone Calls	29	5.1	59	2.9	33	4.2		
Play Games	78	13.6	219	10.8	85	10.9		
SMS	9	1.6	36	1.8	12	1.5		
Chat online	102	17.8	322	15.9	138	17.8		
Check emails	5	0.9	5	0.2	3	0.4		
Watch videos/movies	30	5.2	100	4.9	35	4.5		
Listen to music	30	5.2	103	5.1	33	4.2		
Read news	2	0.3	14	0.7	4	0.5		
Take pictures	4	0.7	9	0.4	2	0.3		
Other	35	6.1	102	5.0	36	4.6		
Survival days without a smartphone	(21.22)	(85.26)	(17.68)	(68.14)	(16.18)	(59.31)	1.44	0.009
Smartphone Addiction	(37.09)	(9.12)	(34.10)	(9.16)	(34.46)	(9.80)	1.68	0.002
Nomophobia	(84.40)	(22.18)	(81.13)	(21.62)	(80.69)	(23.07)	1.08	0.268

^a^ = taken from the actual number of people in the family, provided by the respondents.

**Table 4:** Summary of the multiple linear regression analysis of personal profiles, family environment, smartphone usage patterns, smartphone addiction, and nomophobia (predicting low, average, and high perceived academic performance).

Variables	Low PAP				Average PAP				High PAP			
	B	SE	β	*t*	B	SE	β	*t*	B	SE	β	*t*
**Personal Profiles:**												
Gender	−0.034	0.014	−0.044	−2.489 *	0.075	0.018	0.075	4.208 *	−0.041	0.015	−0.048	−2.688 **
Age	0.017	0.009	0.073	1.997 *	−0.013	0.011	−0.043	−1.166	−0.004	0.010	−0.015	−0.409
Grade level	−0.024	0.011	−0.097	−2.176 *	0.016	0.015	0.050	1.123	0.008	0.013	0.028	0.616
High school level	−0.035	0.025	−0.046	−1.424	0.022	0.032	0.022	0.667	0.014	0.028	0.016	0.483
**Family Environment:**												
Mother’s education	0.006	0.006	0.020	0.931	0.008	0.008	0.021	0.969	−0.013	0.007	−0.043	−1.946
Father’s education	0.012	0.006	0.044	1.994 *	−0.019	0.008	−0.054	−2.454 *	0.007	0.007	0.024	1.089
Parents’ marital status	−0.001	0.003	−0.007	0-.419	0.004	0.004	0.016	0.920	−0.003	0.004	−0.012	−0.698
Family income type	−0.024	0.013	−0.031	−1.799	0.008	0.017	0.008	0.456	0.016	0.015	0.019	1.058
Family size	0.000	0.004	−0.001	−0.035	−0.010	0.005	−0.054	−2.114 *	0.010	0.004	0.064	2.484 *
**Smartphone Usage Patterns:**												
Time until first smartphone use	−0.007	0.006	−0.018	−1.069	−0.002	0.008	−0.003	−0.189	0.008	0.007	0.020	1.163
Time spent per day (on weekdays)	0.013	0.008	0.037	1.589	−0.001	0.001	−0.029	−1.564	0.001	0.001	0.033	1.760
Time spent per day (on weekends)	0.001	0.002	0.014	0.548	0.001	0.002	0.011	0.416	−0.002	0.002	−0.025	−0.966
Frequency of use (on weekdays)	−0.003	0.001	−0.111	−2.423 *	0.002	0.002	0.061	1.304	0.001	0.001	0.029	0.624
Frequency of use (on weekends)	0.001	0.000	0.046	1.826	0.000	0.000	−0.013	−0.533	0.000	0.000	−0.025	−0.992
Years of smartphone experience	−0.005	0.007	−0.036	−0.723	−0.002	0.009	−0.014	−0.268	0.008	0.008	0.048	0.950
Type of internet access	−0.011	0.006	−0.031	−1.741	0.025	0.008	0.054	3.041 **	−0.014	0.007	−0.035	−1.994 *
Purpose of use	0.006	0.002	0.048	2.815 **	−0.003	0.003	−0.017	−0.975	−0.003	0.003	−0.023	−1.352
Survival days without a smartphone	0.000	0.000	0.063	2.834 **	0.000	0.006	0.001	0.032	0.000	0.000	−0.055	−2.452 *
**Smartphone Addiction**	0.005	0.001	0.126	5.591 ***	−0.005	0.001	−0.101	−4.473 ***	0.000	0.001	0.006	0.259
**Nomophobia**	−0.048	0.000	−0.004	−0.184	0.001	0.001	0.024	1.065	0.000	0.000	−0.025	−1.074

Low PAP: (R: 0.202, R^2^ = 0.041, ΔR^2^ = 0.033; F: 5.257; *p* < 0.01) Average PAP: (R: 0.154, R^2^ = 0.024, ΔR^2^ = 0.016; F: 2.997; *p* < 0.01) High PAP: (R: 0.135, R^2^ = 0.018, ΔR^2^ = 0.010; F: 2.318; *p* < 0.01) * *p* < 0.05, ** *p* < 0.01, *** *p* < 0.001.

## Data Availability

The data presented in this study are available on request from the corresponding author. The data are not publicly available due to confidentiality.

## References

[1] Samaha M., Hawi N.S. (2016). Relationships among smartphone addiction, stress, academic performance, and satisfaction with life. Comput. Hum. Behav..

[2] Haug S., Castro R.P., Kwon M., Filler A., Kowatsch T., Schaub M.P. (2015). Smartphone use and smartphone addiction among young people in Switzerland. J. Behav. Addict..

[3] Kwon M., Lee J.Y., Won W.Y., Park J.W., Min J.A., Hahn C., Gu X., Choi J.H., Kim D.J. (2013). Development and validation of a Smartphone Addiction Scale (SAS). PLoS ONE.

[4] Lemola S., Perkinson-Gloor N., Brand S., Dewald-Kaufmann J.F., Grob A. (2015). Adolescents’ electronic media use at night, sleep disturbance, and depressive symptoms in the smartphone age. J. Youth Adolesc..

[5] Tamura H., Nishida T., Tsuji A., Sakakibara H. (2017). Association between excessive use of mobile phone and insomnia and depression among Japanese adolescents. Int. J. Environ. Res. Public Health.

[6] Ahmed R.R., Salman F., Malik S.A., Streimikiene D., Soomro R.H., Pahi M.H. (2020). Smartphone use and academic performance of university students: A mediation and moderation analysis. Sustainability.

[7] Amez S., Vujic S., De Marez L., Baert S. Smartphone Use and Academic Performance: First Evidence from Longitudinal Data. https://ssrn.com/abstract=3521679.

[8] Amez S., Baert S. (2020). Smartphone use and academic performance: A literature review. Int. J. Educ. Res..

[9] Baert S., Vujić S., Amez S., Claeskens M., Daman T., Maeckelberghe A., Omey E., De Marez L. (2020). Smartphone use and academic performance: Correlation or causal relationship?. Kyklos.

[10] Chaudhury P., Tripathy H.K. (2018). A study on impact of smartphone addiction on academic performance. Int. J. Eng. Technol..

[11] Felisoni D.D., Godoi A.S. (2018). Cell phone usage and academic performance: An experiment. Comput. Educ..

[12] Grant J.E., Lust K., Chamberlain S.R. (2019). Problematic smartphone use associated with greater alcohol consumption, mental health issues, poorer academic performance, and impulsivity. J. Behav. Addict..

[13] Hawi N.S., Samaha M. (2016). To excel or not to excel: Strong evidence on the adverse effect of smartphone addiction on academic performance. Comput. Educ..

[14] Kim M.H., Min S., Ahn J.S., An C., Lee J. (2019). Association between high adolescent smartphone use and academic impairment, conflicts with family members or friends, and suicide attempts. PLoS ONE.

[15] Nayak J.K. (2018). Relationship among smartphone usage, addiction, academic performance and the moderating role of gender: A study of higher education students in India. Comput. Educ..

[16] Omer O.Z.E.R. (2020). Smartphone addiction and fear of missing out: Does smartphone use matter for students’ academic performance?. J. Comput. Educ. Res..

[17] Yildiz Durak H.Y. (2019). Investigation of nomophobia and smartphone addiction predictors among adolescents in Turkey: Demographic variables and academic performance. Soc. Sc. J..

[18] Ng S.F., Hassan N.S.I.C., Nor N.H.M., Malek N.A.A. (2017). The relationship between smartphone use and academic performance: A case of students in a Malaysian tertiary institution. Malays. Online J. Educ. Technol..

[19] Kibona L., Mgaya G. (2015). Smartphones’ effects on academic performance of higher learning students. J. Multidiscip. Eng. Sci. Technol..

[20] Yi Y.J., You S., Bae B.J. (2016). The influence of smartphones on academic performance: The development of the technology-to-performance chain model. Library Hi Tech.

[21] Troll E.S., Friese M., Loschelder D.D. (2020). How students’ self-control and smartphone-use explain their academic performance. Comput. Hum. Behav..

[22] Han S., Yi Y.J. (2019). How does the smartphone usage of college students affect academic performance?. J. Comput. Assist. Learn..

[23] Lin T.T., Chiang Y.H. (2017). Investigating predictors of smartphone dependency symptoms and effects on academic performance, improper phone use and perceived sociability. Int. J. Mob. Commun..

[24] Giunchiglia F., Zeni M., Gobbi E., Bignotti E., Bison I. (2018). Mobile social media usage and academic performance. Comput. Hum. Behav..

[25] Yıldız Durak H.Y. (2019). What would you do without your smartphone? Adolescents’ social media usage, locus of control, and loneliness as a predictor of nomophobia. Addicta Turk. J. Addict..

[26] Bian M., Leung L. (2015). Linking loneliness, shyness, smartphone addiction symptoms, and patterns of smartphone use to social capital. Soc. Sci. Comput. Rev..

[27] Emanuel R., Bell R., Cotton C., Craig J., Drummond D., Gibson S., Harris A., Harris M., Hatcher-Vance C., Jones S. (2015). The truth about smartphone addiction. Coll. Stud. J..

[28] SecurEnvoy 66% of the Population Suffer from Nomophobia the Fear of Being without Their Phone. http://www.securenvoy.com/blog/2012/02/16/66-of-the-population-suffer-fromnomophobia-the-fear-of-being-without-their-phone/.

[29] Yildirim C. (2014). Exploring the Dimensions of Nomophobia: Developing and Validating a Questionnaire Using Mixed Methods Research. Master’s Thesis.

[30] Buctot D.B., Kim N., Park K.E. (2018). Development and evaluation of smartphone detox program for university students. Int. J. Contents.

[31] Byeon H.S. (2017). The review of needs and benefits of digital detox. Kor. Man. Consult. Rev..

[32] Griffiths M.D. (2005). A components model of addiction within a biopsychosocial framework. J. Subst. Use.

[33] Goswami V., Singh D.R. (2016). Impact of mobile phone addiction on adolescent’s life: A literature review. Int. J. Home Sci..

[34] Gurbuz I.B., Ozkan G. (2020). What is your level of nomophobia? An investigation of prevalence and level of nomophobia among young people in Turkey. Commun. Ment. Health J..

[35] Güzel Ş. (2018). Fear of the age: Nomophobia (No-Mobile-Phone). J. Acad. Perspect. Soc. Stud..

[36] Gezgin D.M., Şumuer E., Arslan O., Yildirim S. (2017). Nomophobia prevalence among pre-service teachers: A case of Trakya University. Trakya Üniversitesi Eğitim Fakültesi Dergisi.

[37] Lucas D.L. Using Smartphones among Filipinos’ Top Daily Activities. http://business.inquirer.net/183389/using-smartphones-Among-filipinos-top-daily-activities#ixzz5JLd1GHex/.

[38] Sanchez M.J. Smartphone Users in the Philippines 2017. https://www.statista.com/statistics/467186/forecast-of-smartphone-users-in-the-philippines/.

[39] Buctot D.B., Kim N., Kim J.J. (2020). Factors associated with smartphone addiction prevalence and its predictive capacity for health-related quality of life among Filipino adolescents. Child. Youth Serv. Rev..

[40] Roa A. One of 3 Filipinos Can’t Live without Cell Phones—Survey. http://technology.inquirer.net/18168/one-of-3-filipinos-cant-live-without-cell-phones-survey#ixzz5JLeeJKWO.

[41] DepEd News DepEd Order Prohibits the Use of Cell Phones during Class Hours. https://www.depedclick.cf/2019/04/deped-order-prohibits-use-of-cell.html.

[42] Van Deursen A.J., Bolle C.L., Hegner S.M., Kommers P.A. (2015). Modeling habitual and addictive smartphone behavior: The role of smartphone usage types, emotional intelligence, social stress, self-regulation, age, and gender. Comput. Hum. Behav..

[43] Roberts J.A., Yaya L.H., Manolis C. (2014). The invisible addiction: Cell-phone activities and addiction among male and female college students. J. Behav. Addict..

[44] Smetaniuk P. (2014). A preliminary investigation into the prevalence and prediction of problematic cell phone use. J. Behav. Addict..

[45] Buctot D.B., Kim N., Kim S.H. (2020). The role of nomophobia and smartphone addiction in the lifestyle profiles of junior and senior high school students in the Philippines. Soc. Sci. Humanit. Open.

[46] Tran D. (2016). Classifying nomophobia as smart-phone addiction disorder. UC Merced Undergrad. Res. J..

[47] Kim H.J., Min J.Y., Min K.B., Lee T.J., Yoo S. (2018). Relationship among family environment, self-control, friendship quality, and adolescents’ smartphone addiction in South Korea: Findings from nationwide data. PLoS ONE.

[48] Dinc M. (2015). Technology dependence and youth. J. Youth Stud..

[49] Soharwardi M.A., Fatima A., Nazir R., Firdous A. (2020). Impact of parental socioeconomic status on academic performance of students: A case study of Bahawalpur, Pakistan. J. Econ. Econ. Educ. Res..

[50] Boateng S., Asare D., Manu P.T., Sefah E.A., Adomako J. Relationship between students’ home background and their academic performance: A case of some selected senior high school students in rural districts in Ashanti Region, Ghana. J. Educ..

[51] Park H., Lee K.S. (2020). The association of family structure with health behavior, mental health, and perceived academic achievement among adolescents: A 2018 Korean nationally representative survey. BMC Public Health.

[52] Gezgin D.M., Cakir O., Yildirim S. (2018). The relationship between levels of nomophobia prevalence and internet addiction among high school students: The factors influencing nomophobia. Int. J. Res. Educ. Sci..

[53] Jilisha G., Venkatachalam J., Menon V., Olickal J.J. (2019). Nomophobia: A mixed-methods study on prevalence, associated factors, and perception among college students in Puducherry, India. Indian J. Psychol. Med..

[54] Lee H., Ahn H., Choi S., Choi W. (2014). The SAMS: Smartphone Addiction Management System and verification. J. Med. Syst..

[55] Lin Y.H., Lin Y.C., Lee Y.H., Lin P.H., Lin S.H., Chang L.R., Tseng H.W., Yen L.Y., Yang C.C., Kuo T.B. (2015). Time distortion associated with smartphone addiction: Identifying smartphone addiction via a mobile application (App). J. Psychiatr. Res..

[56] Argumosa-Villar L., Boada-Grau J., Vigil-Colet A. (2017). Exploratory investigation of theoretical predictors of nomophobia using the Mobile Phone Involvement Questionnaire (MPIQ). J. Adolesc..

[57] Von Stumm S., Hell B., Premuzic T. (2011). The hungry mind: Intellectual curiosity is the third pillar of academic performance. Perspect. Psychol. Sci..

[58] Jayanthi S.V., Balakrishnan S., Ching A.L.S., Latiff N.A.A., Nasirudeen A.M.A. (2014). Factors contributing to academic performance of students in a tertiary institution in Singapore. Am. J. Educ. Res..

[59] Garton B.L., Ball A.L., Dyer J.E. (2002). The academic performance and retention of college of agriculture students. J. Agric. Educ..

[60] Kennett D.J., Reed M.J. (2009). Factors influencing academic success and retention following a 1st-year post-secondary success course. Educ. Res. Eval..

[61] Lei P.W., Bassiri D., Schultz E.M. (2001). Alternatives to the Grade Point Average as a Measure of Academic Achievement in College.

[62] Clarkson R.K. Effect of Gratitude on Life Satisfaction and Perceived Academic Performance in Psychology Students. Master’s Thesis.

[63] Bandura A. (1989). Human agency in social cognitive theory. Am. Psychol..

[64] Davis R.A. (2001). A cognitive-behavioral model of pathological internet use. Comput. Hum. Behav..

[65] Kwon M., Kim D.J., Cho H., Yang S. (2013). The Smartphone Addiction Scale: Development and validation of a short version for adolescents. PLoS ONE.

[66] IBM (2015). SPSS Statistics for Windows, Version 23.0.

[67] Schreiber-Gregory D. Logistic and linear regression assumptions: Violation recognition and control. Proceedings of the 26th SESUG Conference.

[68] Ghasemi A., Zahediasi S. (2012). Normality tests for statistical analysis: A guide for non-statisticians. Int. J. Endocrinol. Metab..

[69] Coakes S.J. (2005). SPSS: Analysis without Anguish: Version 12.0 for Windows.

[70] Sadler-Smith E. (1996). Approaches to studying: Age, gender and academic performance. Educ. Stud..

[71] Momanyi J.M., Too J., Simiyu C. (2015). Effect of students’ age on academic motivation and academic performance among high school students in Kenya. Asian J. Educ. E-learn..

[72] Liu X., Luo Y., Liu Z.Z., Yang Y., Liu J., Jia C.X. (2020). Prolonged mobile phone use is associated with poor academic performance in adolescents. Cyberpsychol. Behav. Soc. Netw..

[73] Woodcock B., Middleton A., Nortcliffe A. (2012). Considering the Smartphone Learner: An investigation into student interest in the use of personal technology to enhance their learning. Stud. Engagem. Exp. J..

[74] Zhao J., Yuping W., Maideen I., Moe Z.K., Nasirudeen A.M.A. (2018). The relationship between smartphone use and academic performance in a sample of tertiary students in Singapore: A cross-sectional study. J. Educ. Technol..

[75] Junco R., Heiberger G., Loken E. (2011). The effect of twitter on college student engagement and grades. J. Comput. Assist. Learn..

[76] Junco R. (2012). The relationship between frequency of Facebook use, participation in Facebook activities, and student engagement. Comput. Educ..

[77] Gezgin D.M. (2018). Understanding patterns for smartphone addiction: Age, sleep duration, social network use and fear of missing out. Cypriot J. Educ. Sci..

[78] Andreassen C.S., Pallesen S., Griffiths M.D. (2017). The relationship between addictive use of social media, narcissism, and self-esteem: Findings from a large national survey. Addict. Behav..

[79] Masood A., Luqman A., Feng Y., Ali A. (2020). Adverse consequences of excessive social networking site use on academic performance: Explaining underlying mechanism from stress perspective. Comput. Hum. Behav..

[80] Liu D., Kirschner P.A., Karpinski A.C. (2017). A meta-analysis of the relationship of academic performance and Social Network Site use among adolescents and young adults. Comput. Hum. Behav..

[81] Rodríguez-García A.M., Moreno-Guerrero A.J., López Belmonte J. (2020). Nomophobia: An individual’s growing fear of being without a smartphone—A systematic literature review. Int. J. Environ. Res. Public Health.

[82] Qutishat M., Lazarus E.R., Razmy M., Packianathan S. (2020). University students’ nomophobia prevalence, sociodemographic factors and relationship with academic performance at a university in Oman. Int. J. Africa Nurs. Sci..

[83] Bjerre-Nielsen A., Andersen A., Minor K., Lassen D.D. (2020). The negative effect of smartphone use on academic performance may be overestimated: Evidence from a 2-year panel study. Psychol. Sci..

[84] Adelantado-Renau M., Moliner-Urdiales D., Cavero-Redondo I., Beltran-Valls M.R., Martínez-Vizcaíno V., Álvarez-Bueno C. (2019). Association between screen media use and academic performance among children and adolescents: A systematic review and meta-analysis. JAMA Pediatr..

[85] Wajcman J., Bittman M., Jones P., Johnstone L., Brown J. (2007). The Impact of the Mobile Phone on Work/Life Balance. Survey Report.

[86] Rozgonjuk D., Sindermann C., Elhai J.D., Montag C. (2020). Fear of missing out (FOMO) and social media’s impact on daily-life and productivity at work: Do WhatsApp, Facebook, Instagram, and Snapchat use disorders mediate that association?. Addict. Behav..

[87] Kuśnierz C., Rogowska A.M., Pavlova I. (2020). Examining gender differences, personality traits, academic performance, and motivation in Ukrainian and Polish students of physical education: A cross-cultural study. Int. J. Environ. Res. Public Health.

[88] Amoo T.B., Adeyinka O.P., Aderibigbe A.D. (2018). Perceived effects of parental socio-economic status on students’ academic performance among teachers in Odeda Local Government, Ogun State, Nigeria. Int. J. Acad. Res. Bus. Soc. Sci..

[89] Tam V.C. (2009). A comparison of fathers’ and mothers’ contributions in the prediction of academic performance of school-age children in Hong Kong. Int. J. Psychol..

[90] Nora A., Snyder B.P. (2008). Technology and higher education: The impact of e-learning approaches on student academic achievement, perceptions and persistence. J. Coll. Stud. Ret..

